# Efficacy of Mulligan Mobilization, Maitland Mobilization, High-Velocity Thrust, and Muscle Energy Technique for Pain, Disability, and Center-of-Gravity Displacement in Patients With Sacroiliac Joint Dysfunction: Protocol for a Randomized Controlled Trial

**DOI:** 10.2196/87715

**Published:** 2026-08-21

**Authors:** Neha Chitale, Lajwanti Lalwani

**Affiliations:** 1 Ravi Nair Physiotherapy College, Datta Meghe Institute of Higher Education and Research, Wardha, Maharashtra, 442107, India, 91 8605234269

**Keywords:** sacroiliac joint, disability, pain, center of gravity, manual therapy, Mulligan mobilization, Maitland mobilization, high-velocity thrust, muscle energy technique, physiotherapy

## Abstract

**Background:**

Sacroiliac joint (SIJ) dysfunction is associated with significant pain, disability, and altered postural control in adults. Manual therapies, including Mulligan mobilization, Maitland mobilization, high-velocity thrust (HVT), and muscle energy technique (MET), are routinely applied for the management of SIJ dysfunction. However, comparative evidence regarding their effects on pain, disability, and center-of-gravity (COG) displacement remains insufficient.

**Objective:**

This study aims to compare the efficacy of Mulligan mobilization, Maitland mobilization, HVT, and MET in improving pain, functional disability, and COG displacement in individuals with SIJ dysfunction.

**Methods:**

A randomized controlled, parallel-group trial will be conducted on participants diagnosed with unilateral SIJ dysfunction. Participants will be assigned in equal numbers to 4 groups, each receiving Mulligan mobilization, Maitland mobilization, HVT, or MET interventions administered 5 days per week for 3 weeks, in addition to a standardized exercise regimen. The primary outcomes will be pain (assessed using a visual analog scale [VAS]) and functional disability (assessed using the Modified Oswestry Disability Index [MODI]), and the secondary outcome will be COG displacement (measured by sway velocity, lift-up index on the left and right, COG alignment, movement time, and impact index).

**Results:**

We hypothesize that all interventions will lead to statistically significant reductions in pain and disability. Results will be analyzed after the completion of the study.

**Conclusions:**

This protocol is designed to provide robust comparative data on manual therapy approaches for SIJ dysfunction. Findings may guide physiotherapy practice by informing evidence-based recommendations for optimal management strategies.

## Introduction

The sacroiliac joint (SIJ) is a diarthrodial synovial joint that connects the spine to the lower extremities. Although it has limited mobility, the SIJ provides essential stability and plays a significant role in pelvic biomechanics. There are two SIJs, each located between the sacrum and the ilia on either side of the body; together with the sacrococcygeal joint and the pubic symphysis, the SIJs form the pelvic girdle [1]. The primary function of the SIJ is to bear and transfer the weight of the axial skeleton to the hip bones. This weight is then distributed to the femurs when standing or to the ischial bones when seated.

SIJ dysfunction is a condition characterized by pain and reduced range of motion of the SIJ, which may present as generalized low back pain. Studies suggest that SIJ dysfunction has a prevalence rate of 15% to 30% among individuals with low back pain [2]. The SIJ is a key component of the pelvis, and SIJ dysfunction can therefore lead to functional disability [3]. The pain and decreased range of motion associated with SIJ dysfunction make daily activities difficult. Chronic SIJ dysfunction can also displace the body’s center of gravity (COG), which typically passes through the second sacral vertebra (S2); this may further affect weight distribution and lead to gait abnormalities.

Various treatments for SIJ dysfunction, including Mulligan mobilization, Maitland mobilization, and conventional physiotherapy approaches [4], aim to alleviate pain and disability in patients with SIJ dysfunction, particularly those with long-term dysfunction. Chronic issues in the sacroiliac region can result in biomechanical alterations, leading to a shift in the COG and associated functional impairments. A 2016 systematic review of SIJ dysfunction examined treatment approaches but excluded manual therapy techniques [5]. This highlights a gap in the existing literature on the role of manual therapy in managing SIJ dysfunction.

Currently, there is insufficient evidence to guide practitioners in selecting the most effective techniques for treating SIJ dysfunction. While studies have investigated the effects of Mulligan mobilization, Maitland mobilization, high-velocity thrust (HVT), and conventional physiotherapy on pain and functional disability in SIJ dysfunction, findings remain inconclusive. Available evidence is conflicting regarding which intervention is most effective, creating uncertainty among practitioners.

Although each technique is well established individually, no comparative study has been conducted to evaluate the relative efficacy of these manual therapy approaches. A comparative analysis of these techniques could provide valuable evidence to guide clinical decision-making and optimize treatment outcomes for individuals with SIJ dysfunction.

This study aims to compare the efficacy of Mulligan mobilization, Maitland mobilization, HVT, and muscle energy technique (MET) in improving pain, functional disability, and COG displacement in patients with SIJ dysfunction and to rank these approaches according to their efficacy.

## Methods

### Ethical Considerations

Following approval from the Datta Meghe Institute of Higher Education and Research Institutional Ethics Committee on April 9, 2025 (DMIHER(DU)/IEC/2025/230), 108 participants will be recruited through the Department of Musculoskeletal Physiotherapy at Ravi Nair Physiotherapy College, Sawangi (Meghe), Wardha. All participants will provide written informed consent before enrollment and will be given the opportunity to ask the researcher questions. Participants may withdraw from the study at any time. This trial is registered with Clinical Trials Registry–India (CTRI/2025/08/093085).

### Study Design

This is a prospective, 4-arm, parallel-group, randomized controlled trial to assess superiority. The study protocol was developed in accordance with the SPIRIT (Standard Protocol Items: Recommendations for Interventional Trials) guidelines. Data will be collected on pain, disability, and COG displacement. The trial will be reported in accordance with the CONSORT (Consolidated Standards of Reporting Trials) 2010 guidelines.

### Eligibility Criteria

Inclusion criteria are as follows: participants must (1) be willing to sign informed consent, (2) be diagnosed with SIJ dysfunction, (3) be aged 18 to 35 years, (4) have positive Gaenslen and Gillet tests, (5) have a symptom duration of 4 to 8 weeks, and (6) have a BMI of 18.5 to 25. Participants will be excluded if they (1) have an active infection, (2) have a cognitive disorder, (3) have osteoporosis, (4) have a history of recent surgery, (5) have posttraumatic back pain, or (6) have lumbar radiculopathy.

### Participant Recruitment

Participants who meet the inclusion criteria will be enrolled in this study. A computer-generated randomization sequence will be used to randomly allocate participants to 4 groups in a 1:1:1:1 ratio. The sequence will be generated by a supervisor (LL) who will not be involved in recruitment or treatment. The allocation sequence will be placed in sequentially numbered, opaque, sealed envelopes by the supervisor. The envelopes will be stored in a locked cabinet until they are opened by the treating therapist immediately prior to treatment. Participants will be blinded to group allocation and the specific manual therapy technique received. All interventions will be delivered in the same clinical setting with standardized session duration. Outcomes will be assessed by an independent assessor who will be blinded to treatment allocation. The treating therapist will not be involved in outcome assessment. Unblinding will not be permitted for the participants or assessor.

### Sample Size and Variables

The total sample size will be 108 participants (27 in each of 4 groups).

The independent variables include Mulligan mobilization, Maitland mobilization, HVT, and MET. The dependent variables include pain (measured by the VAS), functional disability (measured by the MODI), and COG displacement (measured using a force plate; measurements will include sway velocity, lift-up index on the left and right, COG alignment, movement time, and impact index). Cointerventions include lumbar stabilization exercises and the application of hot moist packs.

### Procedure

Patients with low back pain presenting to Acharya Vinoba Bhave Rural Hospital, Sawangi (Meghe), Wardha, or the Musculoskeletal Outpatient Department, Ravi Nair Physiotherapy College, will be assessed for SIJ dysfunction, and their diagnosis will be confirmed through investigations and physical examination following approval from the institution’s ethics committee. Patients who meet all inclusion criteria and none of the exclusion criteria will be invited to participate in this study. In total, 108 participants will be enrolled after providing informed written consent. Demographic information will be gathered, and the baseline VAS score, MODI score, and COG displacement will be assessed for each participant. Then, participants will be randomized into 4 groups: group A (Mulligan mobilization), group B (Maitland mobilization), group C (HVT), and group D (MET). Each group will receive the assigned treatment 5 days per week for 3 weeks [6-8]. After 3 weeks of treatment, a postintervention evaluation will be conducted, and VAS scores, MODI scores, and COG displacement will be reassessed. These outcomes will also be reassessed two months after the intervention, at which point the data will be collected for analysis (see Textbox 1).

Textbox 1. **Steps at each stage of the study.**

**Prescreening**

- Informed consent collected
- Patients assessed by inclusion and exclusion criteria

**Baseline assessment (T0)**

- Demographic information collected
- Patients further assessed by inclusion and exclusion criteria
- Participants selected and randomized into 4 groups
- Pain assessed (visual analog scale [VAS])
- Disability assessed (Modified Oswestry Disability Index [MODI])
- Center-of-gravity (COG) displacement assessed (sway velocity, lift-up index, COG alignment, movement time, and impact index)
- Adverse events monitored for

**Intervention, 5 sessions per week for 3 weeks (T1)**

- Intervention performed (moist heat treatment, manual therapy, lumbar stabilization exercises)
- Compliance check performed

**Postintervention assessment immediately after final intervention session (T2)**

- Pain assessed (VAS)
- Disability assessed (MODI)
- Cog displacement assessed (sway velocity, lift-up index, COG alignment, movement time, and impact index)
- Adverse events monitored for
- Compliance check performed

**Follow-up, 2 months after final intervention session (T3)**

- Pain assessed (VAS)
- Disability assessed (MODI)
- COG displacement assessed (sway velocity, lift-up index, COG alignment, movement time, and impact index)
- Adverse events monitored for

### Intervention

This study is designed to evaluate the therapeutic impact of different intervention protocols in patients with SIJ dysfunction. The study will include 4 intervention groups, each receiving conventional moist heat therapy, a manual therapy technique specific to each group, and lumbar stabilization exercises. Treatment sessions for all groups are designed to optimize outcomes and will occur 5 days per week for 3 weeks.

At the start of each session, participants in all groups will receive an identical moist heat treatment via commercial hot packs placed directly against the back for 10 minutes. Then, participants will receive the assigned manual therapy. Group A will receive Mulligan mobilization [9] to the sacral region, focusing on correcting SIJ alignment. Two distinct approaches will be used based on established protocols. These mobilizations will be delivered in 3 sets of 10 repetitions, with a 1-minute rest between sets. Group B will receive Maitland mobilization [8] in 3 sets of 30 seconds each. For the first 2 weeks, group B participants will receive grade 1 to grade 2 Maitland mobilization, and in the third week, grade 3 Maitland mobilization will be given. Group C will receive HVT [10,11], including anterior and posterior thrusts targeting the ilium, administered with the participant positioned supine or in a side-lying position. Specific maneuvers will also be used to correct up-slip and down-slip of the ilium using rapid, controlled thrusts applied by the clinician to restore joint alignment. Group D will receive MET [12,13] to correct anterior SIJ dysfunction, with the participant lying in a lateral position on the unaffected side with the affected leg flexed to the point of initial tension. A contraction intensity of 20% will be used.

After manual therapy, all groups will receive an identical structured lumbar stabilization exercise regimen [14]. This will comprise stretching exercises targeting muscles in the gluteal area, including the piriformis, gluteus medius, minimus, and maximus muscles, and SIJ self-mobilization techniques. Isometric hip muscle strengthening will be incorporated through exercises such as leg presses against a pillow and lateral leg pushes. Lumbar stabilization will be addressed via quadriceps and hamstring stretches, posterior pelvic tilts, standard back bridges with 1 and 2 legs elevated, and sit-ups. These exercises will be prescribed as 2 sets with a 1-minute rest interval between sets. These exercises will be performed 5 days per week in the clinic setting and twice daily at home.

Across all groups, interventions will be structured to ensure consistency of treatment delivery over the 3-week study period. Moist heat will be applied to facilitate tissue relaxation and enhance blood flow before manual therapy. Each manual therapy method has been selected for its documented efficacy in restoring SIJ mobility and alignment. The stabilization exercises will target muscular balance and postural control to improve functional outcomes, while the home exercise program will reinforce therapy gains outside clinical sessions.

### Criteria for Treatment Termination

Treatment will be terminated under specific circumstances for individual participants in each group. Treatment will be terminated for a participant (1) in group A if the participant is unable to actively participate or follow self-maintenance techniques, (2) in group B if symptoms are aggravated by passive movement, that is, if the participant experiences hyperreactivity to passive movement, (3) in group C if the participant experiences negative neurological change, feelings of fear, or excessive joint movement after HVT manipulation, or (4) in group D if the participant experiences muscle guarding or is unable to perform isometric contractions.

### Outcomes

The primary outcomes are changes in pain and disability. The subjective experience of pain will be measured using the VAS (*r*=0.94; *P*<.001) [15]. Participants will be shown the VAS, a 10-cm scale representing levels of pain, where 0 represents no pain and 10 represents unbearable pain, and asked to indicate their current level of pain. Functional disability will be measured using the MODI (*r*=0.87; *P*<.05) [16,17], a questionnaire that provides an assessment of the effect of low back pain on daily life. The score is calculated on a scale of 0% to 100% based on responses to the questionnaire.

The secondary outcome is change in COG displacement from the baseline, measured using a force plate. This will be measured at baseline, after 3 weeks of treatment, and at the 2-month follow-up using a force plate, which will be calibrated every 6 months by a biomedical technician. The force plate will be connected to a personal computer on which COG analysis software is installed. Participants will be asked to remove their footwear, stand on the force plate with their feet positioned on the designated markings, and distribute their weight equally between both feet. Participants will be asked to stand still for 30 seconds while the force plate measures sway velocity, lift-up index on the left and right, COG alignment, movement time, and impact index.

All outcomes will be assessed before the 3-week treatment begins, after three weeks of treatment, and at follow-up 2 months after treatment ends. The primary end points will be the change in all pain and disability from baseline to the 2-month follow-up, and the secondary end point will be the change in COG displacement from baseline to the 2-month follow-up. Changes in outcomes will be compared among the 4 groups after the 2-month follow-up.

### Data Analysis

Statistical analyses will be carried out using SPSS software (version 25.0; IBM Corp). To determine the significance of the differences between the 4 groups, we will first establish normality and then choose which test to use based on the distribution of the data. We will use mixed-design ANOVA if the data are normally distributed and the Friedman test if the data are not normally distributed. Statistical analysis will use a linear mixed-effects model for group-by-time interaction. Analyses will follow the intention-to-treat principle. Missing data will be handled by the missing-at-random assumption using maximum likelihood estimation. Planned pairwise comparisons will be adjusted for multiple testing using the Holm method. Repeated-measures ANOVA will be used to determine the significance of changes among the baseline, postintervention, and follow-up assessments. Frequency distribution and descriptive statistics will be used to analyze demographic information. For all statistical tests, *P*<.05 will be considered statistically significant, and 95% CIs will be reported.

Data collection and reporting will be performed under the guidance of the principal investigators, and documentation for the analysis will be reviewed for accuracy. An Excel spreadsheet containing the study data will be provided to a statistician who will be blinded to treatment allocation for statistical analysis after study completion. The trial data will be stored in a secure, locked location for later analysis by a biostatistician and the primary researcher (NC).

## Results

**Figure 1.**
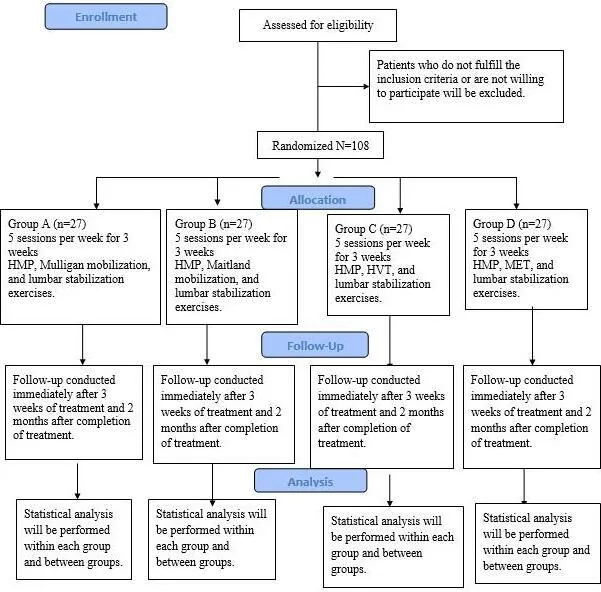
CONSORT (Consolidated Standards of Reporting Trials) 2010 flow diagram. HMP: hot moist pack; HVT: high-velocity thrust; MET: muscle energy technique.

Participant recruitment began in August 2025, and as of November 2025, 63 participants have been recruited. All participants will be recruited by August 2026, after which the intervention will be conducted. We expect to conclude the follow-up assessment by October 2026. Results will be discussed in a follow-up article. Results will be reported in accordance with CONSORT 2010 guidelines; a planned participant flow diagram is shown in Figure 1.

## Discussion

### Primary Findings

We hypothesize that all 4 groups will experience significant reductions in both pain and functional disability. It is expected that COG alignment will also improve after the intervention. The interventions in this study will target distinct pathophysiological elements of SIJ-related complaints. Altered arthrokinematics is a leading cause of SIJ dysfunction, which mobilization may target; mechanical restriction and compensatory motor patterns may also be addressed.

The 4 manual therapy methods have different mechanisms, but reduction in VAS scores is anticipated in all groups. This hypothesis differs from the findings of Trager et al [18], who concluded in a systematic review that manual therapy did not significantly reduce pain. Mulligan mobilization helps to relieve pain linked with bone alignment abnormalities [19] and works on the principle of sustained accessory glide with active movement, which helps to restore pain-free joint tracking. This approach may be used to address postural control and asymmetric intra-articular translation [20,21]. Maitland mobilization is an oscillatory graded mobilization, which helps to reduce hypomobility and irritability of the SIJ [22] along with associated pain and disability. HVT induces cavitation-mediated neurophysiological responses and proprioceptive input from the SIJ, and it facilitates kinematic adjustments [23-25]. MET involves voluntary isometric contractions followed by stretching and uses postisometric relaxation and autogenic inhibition to lengthen hypertonic musculature surrounding the SIJ [26,27]. The assessment of pain, functional disability, and COG displacement is important in SIJ rehabilitation, as dysfunction leads to functional limitations and impairment. Furthermore, COG displacement is linked with altered neuromuscular control and postural stability, and changes in COG alignment are linked with long-term disability. We hypothesize that 3 weeks of treatment in all 4 groups will reduce COG displacement but will not completely return the COG to a neutral alignment.

Although some participants may have muscular abnormalities or imbalance associated with SIJ dysfunction, these factors will not be targeted by in this intervention. Future studies could investigate the long-term effects of the manual therapy techniques in this study and compare them with other manual therapy techniques.

### Conclusions

This 4-arm, parallel-group randomized controlled trial will establish the comparative efficacy of 4 manual therapy techniques—Mulligan mobilization, Maitland mobilization, HVT, and MET—in patients with SIJ dysfunction. Pain, functional disability, and COG displacement will be assessed as outcomes. The comparative effects of the interventions will be evaluated based on statistical analysis after completion of the intervention and follow-up. We hypothesize that all groups will demonstrate significant reductions in pain, functional disability, and COG displacement.

## Abbreviations

COG: center of gravity
CONSORT: Consolidated Standards of Reporting Trials
HMP: hot moist pack
HVT: high-velocity thrust
MET: muscle energy technique
MODI: Modified Oswestry Disability Index
SIJ: sacroiliac joint
SPIRIT: Standard Protocol Items: Recommendations for Interventional Trials
VAS: visual analog scale
